# Inhibition of the chromatin remodeling factor NURF rescued sterility by a clinic variant of NuRD

**DOI:** 10.1091/mbc.E23-05-0197

**Published:** 2023-12-18

**Authors:** Zijie Shen, Zhengyang Guo, Guangshuo Ou, Wei Li

**Affiliations:** a Tsinghua-Peking Center for Life Sciences, Beijing Frontier Research Center for Biological Structure, McGovern Institute for Brain Research, State Key Laboratory of Membrane Biology, School of Life Sciences and MOE Key Laboratory for Protein Science, and; b School of Medicine, Tsinghua University, Beijing 100084, China; Peking University Health Science Center

## Abstract

The nucleosome remodeling and deacetylase (NuRD) complex is essential for gene expression and cell fate determination, and missense mutations of NuRD caused neurodevelopmental diseases. However, the molecular pathogenesis of clinic NuRD variants is unknown. Here, we introduced a clinic CHD3 (L915F) variant into *Caenorhabditis elegans* homologue LET-418, impairing germline and vulva development and ultimately causing animal sterility. Our ATAC-seq and RNA-seq analyses revealed that this variant generated an abnormal open chromatin structure and disrupted the expression of developmental genes. Through genetic suppressor screens, we uncovered that intragenic mutations, likely renovating NuRD activity, restored animal viability. We also found that intergenic mutations in nucleosome remodeling factor NURF that counteracts NuRD rescued abnormal chromatin structure, gene expression, and animal sterility. We propose that two antagonistic chromatin-remodeling factors coordinate to establish the proper chromatin status and transcriptome and that inhibiting NURF may provide insights for treatment of NuRD mutation-related diseases.

## Abstract (executive summary)

The nucleosome remodeling and deacetylase (NuRD) complex is essential for gene expression and cell fate determination. Missense mutations in NuRD cause neurodevelopmental diseases. The molecular mechanisms of pathogenesis for clinical NuRD variants are unknown.

We introduced a clinical CHD3 (L915F) variant into *Caenorhabditis elegans* homologue LET-418, which impaired germline and vulva development and causing animal sterility. Intragenic suppressor mutations restored animal viability. Intergenic mutations in nucleosome remodeling factor NURF rescued abnormal phenotypes.

These results suggest two antagonistic chromatin-remodeling factors coordinate to establish proper chromatin status and transcriptional profile. Inhibiting NURF may provide insights for treatment of diseases related to NuRD mutations.

## INTRODUCTION

In eukaryotic cells, genetic material, or DNA, is intricately arranged around histones, giving rise to nucleosomes, which serve as the fundamental units of chromatin. This structural organization plays a vital role in compacting DNA within the nuclei and safeguarding the integrity of genetic information. Nonetheless, the existence of nucleosomes poses a hindrance to the interaction between trans-elements and DNA, impeding various DNA-dependent processes such as transcription. Hence, the modulation of chromatin structure provides a basal layer of transcriptional regulation (Clapier *et al.*, 2017). Chromatin-remodeling factors utilize the energy derived from ATP hydrolysis to regulate chromatin structure by employing various mechanisms such as sliding, removing, or reconstructing nucleosomes (Clapier *et al.*, 2017). Through the binding and subsequent release of DNA by two catalytic domains of chromatin-remodeling factors, an “inchworm” mechanism is achieved, allowing for the generation of a “DNA wave” around the nucleosome, thereby leading to the movement of nucleosomes along the DNA strand (Liu *et al.*, 2017; Yan and Chen, 2020).

The nucleosome remodeling and deacetylase (NuRD) complex, which couples chromatin remodeling with histone deacetylase activity, represents one of the key chromatin remodeling complexes essential for gene expression, genome integrity, and cell cycle progression. The chromatin remodeling subcomplex consists of CHD3/4/5 (chromodomain helicase DNA-binding protein 3/4/5) that uses the energy from ATP to reposition nucleosome and multiple associated proteins that regulate the catalytic activity of the ATPase (Hoffmann and Spengler, 2019). NuRD activity undergoes stringent regulation to fine-tune gene expression. Differential assembly of a specific CHD endows NuRD with distinct functions across distinct developmental stages and different types of cells (Nitarska *et al.*, 2016; Hoffmeister *et al.*, 2017). Posttranslational modifications of NuRD components also play an important role in regulating NuRD activity. While methylated MTA1 (metastatic tumor antigen 1), a core-subunit of the NuRD complex, is required for formation of a NuRD repressive complex, demethylated MTA1 recruits coactivator NURF (nucleosome remodeling factor), another pivotal chromatin-remodeling complex, to activate gene transcription (Nair *et al.*, 2013). The NURF complex is the founder member of the imitation switch (ISWI) family chromatin remodeling complexes. *Homo sapiens* NURF are composed of the largest subunit BPTF (bromodomain PHD finger transcription factor), the ISWI ATPase subunit SNF2L and pRBAP46/48 (Alkhatib and Landry, 2011). The NURF complex properly spaces nucleosomes to enhance chromatin access and promote transcription (Clapier *et al.*, 2017; Judd *et al.*, 2021). Thus, NuRD and NURF can function as antagonistic chromatin-remodeling factors to ensure proper regulation of the transcriptome (Nair *et al.*, 2013).

The chromatin-remodeling activity of the NuRD complex has been implicated in the development of the neuronal system, and genetic variations in the chromatin-remodeling subunit of the NuRD complex can give rise to neurodevelopmental disorders. Missense substitutions in CHD4 were found in individuals sharing symptoms including intellectual disability, macrocephaly, hearing loss, distinct facial dysmorphisms, palatal abnormalities, ventriculomegaly, and hypogonadism (Weiss *et al.*, 2016). All the identified CHD4 mutations localized to evolutionarily highly conserved residues, which are predicted to affect ATPase catalytic activity. Similar to CHD4, CHD3 mutations, most of which cluster within the ATPase/helicase motif, have been associated with a distinct syndrome characterized by intellectual disability, macrocephaly, impaired speech, and language skills, as well as distinctive facial features (Snijders Blok *et al.*, 2018). A subset of clinical CHD-3 variants showed impaired ATPase activity or chromatin remodeling capacities in vitro. Notably, CHD3 (L915F) demonstrated hyperactive ATPase activity and chromatin remodeling capacities (Snijders Blok *et al.*, 2018). The clinical CHD variants underscore the critical role of NuRD remodeling activity during neurodevelopment. However, the molecular mechanism by which aberrant remodeling activity leads to impaired neurodevelopment remains unclear.

In this study, we established *Caenorhabditis elegans* models for NuRD-associated neurodevelopmental diseases using CRISPR-Cas9 technology, focusing on the CHD3 (L915F) variant. The mutation corresponding to CHD3 (L915F) in *C. elegans* results in impaired germline and vulva development at 26°C, leading to sterility in *C. elegans*. Assay for Transposase-Accessible Chromatin with high-throughput sequencing (ATAC-seq) analysis of the disease model worms identified an abnormal open chromatin structure affecting developmental genes. Genetic screen identified both intragenic and intergenic suppressor mutations that recover phenotypes of disease model worm. Interestingly, intergenic mutations in antagonistic NURF led to the restoration of proper chromatin structure. These results reveal that the coordination between NuRD and NuRF in chromatin remodeling is essential for animal fertility, which provides insights for the development of potential treatments targeting NuRD-associated developmental disorders.

## RESULTS

### A clinical variant of CHD-3 remodeling factor disrupted fertility in *C. elegans*

**FIGURE 1:**
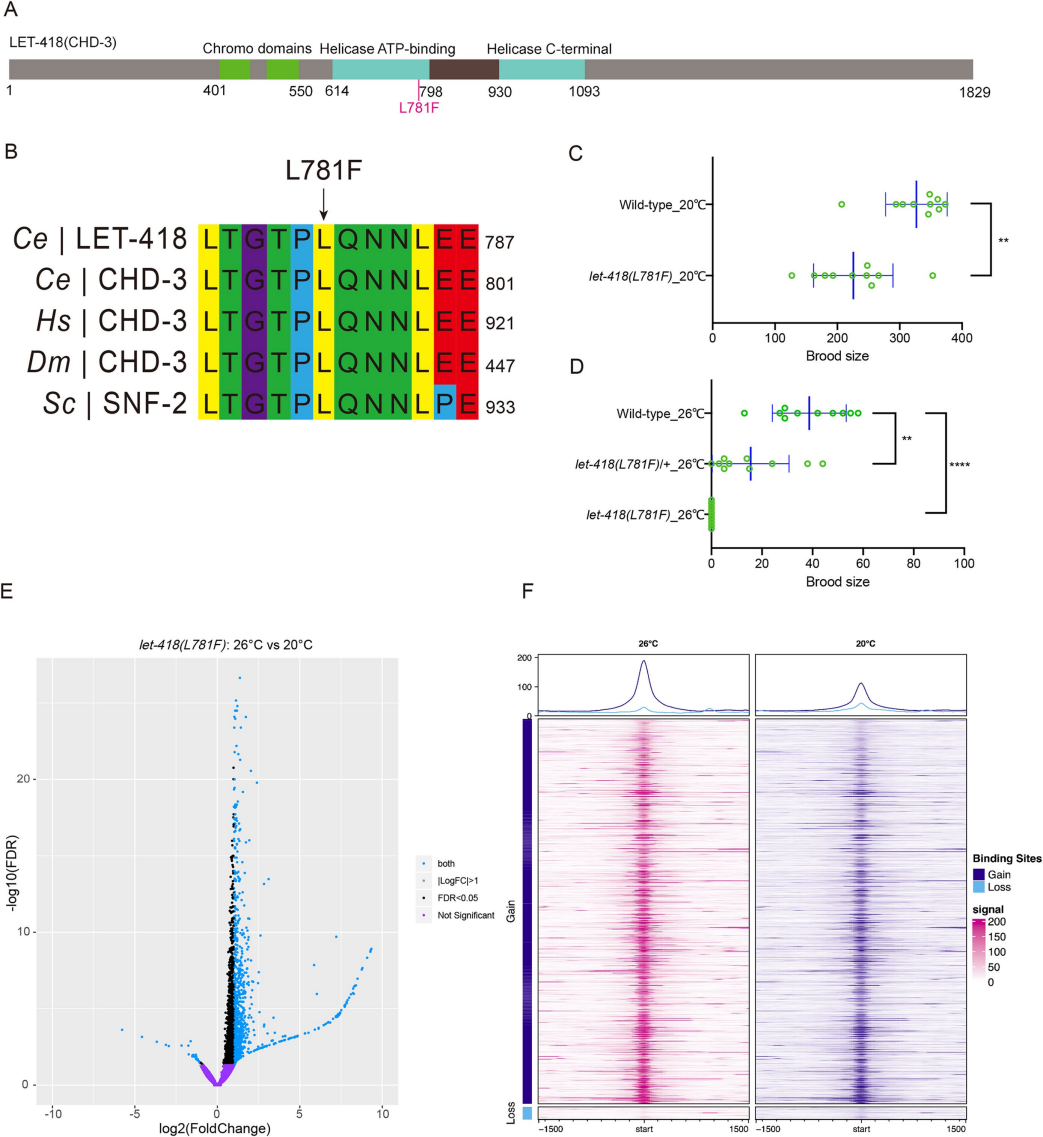
Disease model *let-418(L781F)* worms are sterile at 26°C. (A) Schematic of *C. elegans* LET-418 protein. L781F of LET-418 corresponding to clinical L915F of CHD3 is indicated in red. (B) Multi-sequence alignment of the *C. elegans* (Ce) LET-418 protein and its homologues in worms (*Ce*), human (*Hs*), mouse (Mm), fruit fly (*Dm*), and yeast (*Sc*). Arrow indicates the conserved L781 in LET-418. (C and D) Brood size of wild-type and *let-418(L781F)* animals raised at (C) 20°C and (D) 26°C. *N* = 10, values are presented as mean ± SD (error bars). Statistical significance compared with the control is based on Student’s *t* test, ns, not significant, *p < 0.05, **p < 0.01, ***p < 0.001, ****p < 0.0001. (E) Volcano plot comparing ATAC signals of each gene in *let-418(L781F)* mutant at 20 and 26°C. (F) The average and heatmap of ATAC-seq signals at promoter regions with significant changes (|Fold Change| > 2) in *let-418(L781F)* mutant at 20 and 26°C.

The *C. elegans* genome encodes two CHD3 homologues, *let-418* and *chd-3*, which display partially redundant functions during development (von Zelewsky *et al.*, 2000). To study molecular deficiencies caused by neurodevelopmental disease-associated CHD3 (L915F) mutation, we introduced L781F and L795F into the *let-418* gene and the *chd-3* gene, respectively, using CRISPR-Cas9 (Figure 1, A and B). Homozygous *let-418(L781F)* mutant animals displayed normal morphology, viability, and growth rates at 20°C, albeit with a slightly reduced brood size (Figure 1C). However, when subjected to a restrictive temperature of 26°C, *let-418(L781F)* animals became completely sterile, while the wild-type (WT) strain was still able to lay eggs (Figure 1, C and D; Supplemental Figure S1A). Notably, heterozygous *let-418(L781F)* nematodes manifested a decrease in fertility and brood size at 26°C, thus signifying the partial dominance of the *let-418(L781F)* mutation (Figure 1D; Supplemental Figure S1A). This observation mirrors the autosomal dominant mutation pattern identified in CHD3 mutations related to Snijders Blok-Campeau syndrome. Furthermore, more than 90% *let-418(L781F)* animals developed a protruding vulva phenotype, and about 20% even exhibited multivulva phenotypes at 26°C (Supplemental Figure S1, B and C). In contrast, homozygous *chd-3(L795F)* worms displayed normal morphology, viability, growth rates, and brood size comparable to WT both at 20 and 26°C. *let-418(L781F)* and *chd-3(L795F)* phenocopy their null alleles (von Zelewsky *et al.*, 2000), supporting the functional redundancy between LET-418 and CHD-3 and a predominant role of LET-418 in development. Thus, further analysis was carried out in the *let-418(L781F)* mutant.

To gain insights into the mechanisms by which LET-418 regulates germline development, we constructed an LET-418(L781F)::GFP knockin strain and measured diameter of nuclei of germ cells. In line with the chromatin remodeling activity of LET-418, we found that *let-418(L781F)* mutant animals displayed enlarged germline nuclei at 26°C (Supplemental Figure S1, D and E). We propose that the L781F mutation leads to abnormal chromatin accessibility, which in turn results in sterility and other associated phenotypes. To test this hypothesis, we conducted Assay for Transposase-Accessible Chromatin with high-throughput sequencing (ATAC-seq) to examine changes in chromatin accessibility within embryos of *let-418(L781F)* mutants.

We observed that the raw reads from ATAC-seq were enriched at the transcription start site (TSS; Supplemental Figure S1F), and the peaks identified from the reads exhibited similar enrichment (Supplemental Figure S1G), confirming the high quality of the ATAC-seq data. Comparative analysis of ATAC-seq signals revealed that *let-418(L781F)* embryos at 26°C had 1061 genes with upregulated chromatin accessibility and 27 genes with downregulated chromatin accessibility around the promoter regions compared with embryos at 20°C (Figure 1E). The upregulated genes greatly outnumbered the downregulated genes, indicating that the *let-418(L781F)* mutation induces abnormal open chromatin structure around promoters at 26°C, which might account for the aberrant germline and vulva development in *C. elegans*. When compared with WT at 26°C, the *let-418(L781F)* embryos displayed approximately equal numbers of upregulated and downregulated genes (1390 upregulated genes versus 1460 downregulated genes; Supplemental Figure S1H). Noticeably, the upregulated genes in *let-418(L781F)* embryos at 26°C exhibited more pronounced amplitude of ATAC signals variation around promoters than the downregulated genes (Figure 1F; Supplemental Figure S1I). These results collectively indicate that the *let-418(L781F)* mutation predominantly enhance chromatin accessibility, resulting in differential gene expression profile.

To test whether the changes in chromatin accessibility is correlated with abnormal gene expression, we performed RNA-seq analysis. We compared gene expression levels in *let-418(L781F)* mutant at 26°C with those at 20°C and *let-418(L781F)* mutant with WT at 26°C, and observed a higher number of upregulated genes than downregulated genes in *let-418(L781F)* mutant (Supplemental Figure S2, A and B). This observation is consistent with increased chromatin accessibility found in the ATAC-seq data. Additionally, the presence of a more open chromatin structure around the promoter region corresponded to increased mRNA expression levels (Supplemental Figure S2, C and D). These results suggest that changes in chromatin accessibility contribute to the abnormal gene expression in *let-418(L781F)* mutant, which might lead to the observed abnormalities in germline and vulva development at 26°C.

### Intragenic suppressors restored *let-418(L781F)* fertility

**FIGURE 2:**
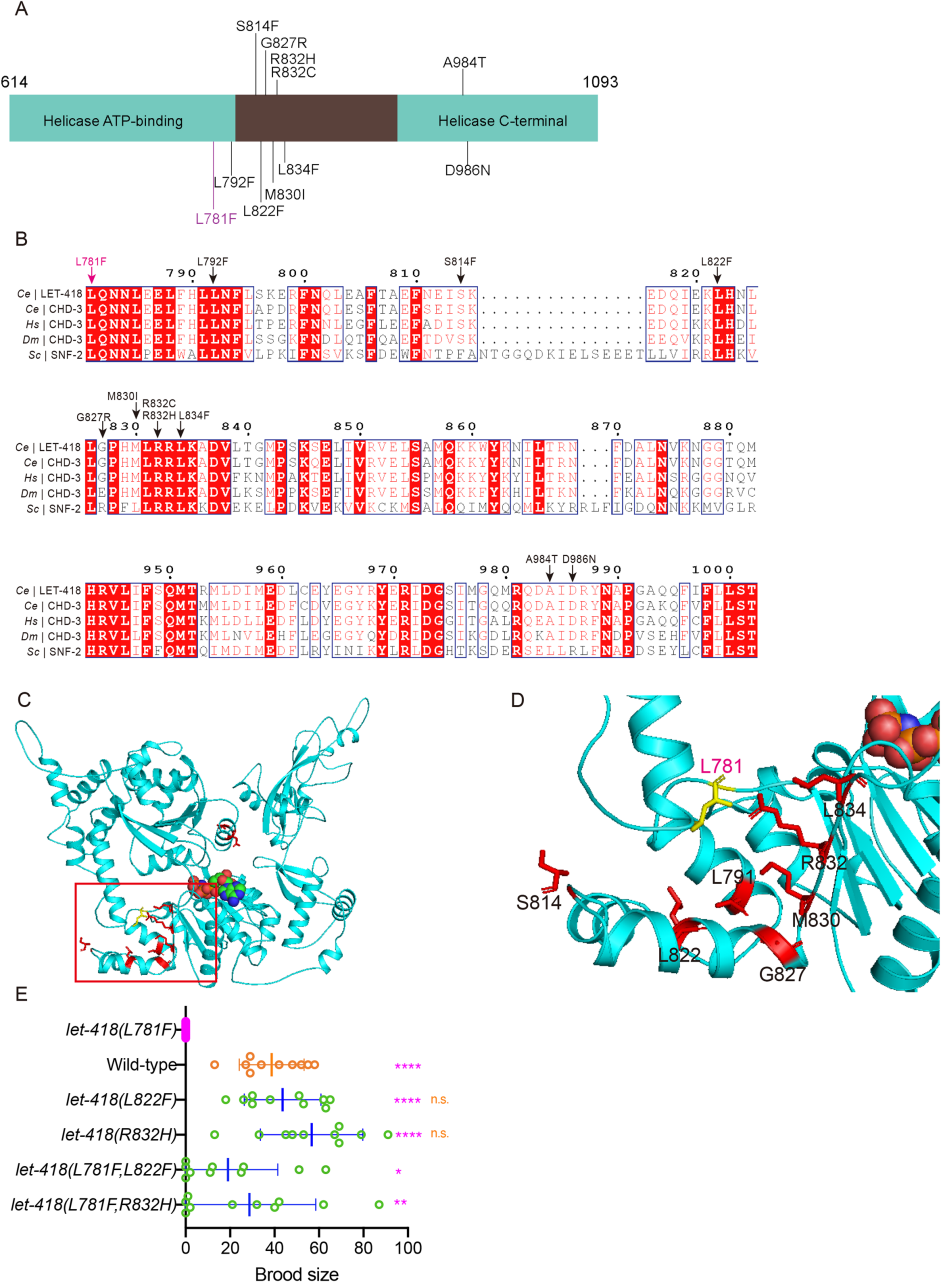
Intragenic mutations restore the fertility of *let-418(L781F)*. (A) Distribution of intragenic suppressors of *let-418(L781F)*. The background mutation L781F is indicated in magenta and suppressor mutations are indicated in black. (B) Multi-sequence alignment of the *C. elegans* (*Ce*) LET-418 protein and its homologues in worms (*Ce*), human (*Hs*), mouse (*Mm*), fruit fly (*Dm*), and yeast (*Sc*). Magenta arrow indicates the background mutation L781F and black arrows indicate suppressor mutations. (C) Alphafold2-prediction of *C. elegans* LET-418 structure with ATP. The background L781F mutation is highlighted in yellow and suppressor mutations are highlighted in red. (D) Zoom-in view of the LET-418 structure boxed in C. (E) Brood size of the indicated strains at 26°C. *N* = 10, values are presented as mean ± SD (error bars). Statistical significance compared with the control with a matching color code is based on Student’s *t* test, ns, not significant, *p < 0.05, **p < 0.01, ***p < 0.001.

To investigate the molecular pathway regulating LET-418 activity, we performed a genetic screen to identify suppressors that recover the fertility in *let-418(L781F)* mutant at 26°C (Supplemental Figure S3A). In total, we isolated 18 independent suppressors of *let-418(L781F)* from ∼50,000 mutagenized haploid genomes. Through whole- genome sequencing and bioinformatics, we determined that 15 suppressor alleles represented 10 intragenic missense mutations (Supplemental Figure S3B). The intragenic mutations were enriched in the region containing the helicase domains of LET-418 and most of residues altered by the suppressor mutations are highly conserved across species (Figure 2, A and B). Based on the predicted structure of LET-418 using AlphaFold2, we observed that seven of the intragenic mutations were close to the background mutation L781, suggesting they might directly restore the structural defects caused by L781F (Figure 2, C and D). The remaining two mutations, A984T and D986N, were relatively distant from the L781F (Figure 2C), suggesting that they might either interact indirectly with L781F or rescue the defects of this clinical variant through interactions with other proteins.

To experimentally validate these identified mutations, we selected two intragenic suppressors, *let-418(L822F)* and *let-418(R832H)*, and introduced the two lesions to WT animals using CRISPR-Cas9. Neither of two mutants exhibited noticeable defects in morphology. Moreover, *let-418(L822F)* and *let-418(R832H)* worms were fertile at 26°C and displayed no significant difference in brood size compared with WT worms (Figure 2E; Supplemental Figure S3C). By introducing *let-418(L822F)* and *let-418(R832H)* mutations into *let-418(L781F)* mutant, we confirmed that they restored the fertility of *let-418(L781F*; Figure 2E; Supplemental Figure S3C). Furthermore, Principal Component Analysis (PCA) of the ATAC-seq data indicated that the chromatin accessibility profiles of *let-418(L781F, L822F)* and *let-418(L781F, R832H)* resembled those of the WT worms rather than *let-418(L781F)* mutant (Supplemental Figure S3D). Together, our results suggest that the intragenic suppressors located near the L781F site might rescue *let-418(L781F)* phenotypes by restoring the remodeling activity of LET-418.

### Mutations in the *nurf-1* gene restored *let-418(L781F)* fertility

**FIGURE 3:**
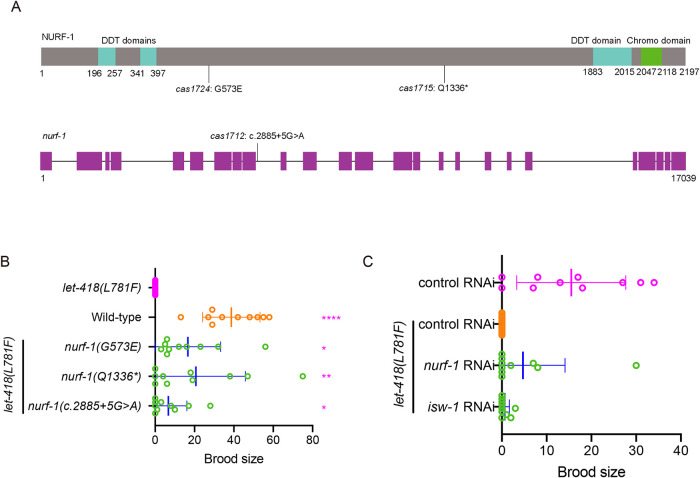
Intergenic mutations in *nurf-1* restore the fertility of *let-418(L781F)*. (A) Schematics of *C. elegans* NURF-1 protein and gene. The missense mutation (*cas1724*), nonsense mutation (*cas1715*) and splicing-region mutation (*cas1712*) are indicated. (B) Brood size of the indicated strains at 26°C. *N* = 10, values are presented as mean ± SD (error bars). Statistical significance compared with the control with a matching color code is based on Student’s *t* test, ns, not significant, *p < 0.05, **p < 0.01, ***p < 0.001. (C) Brood size of the indicated RNAi strains at 26°C. *N* = 10, values are presented as mean ± SD (error bars).

In addition to the intragenic suppressors, we also isolated three intergenic suppressor mutations in the *nurf-1* gene, which encodes an orthologue of the human BPTF in the NURF complex. The three suppressor strains carry a splicing mutation, a premature stop codon (Q1336stop) and a missense mutation (G573E) in the *nurf-1* gene, respectively (Figure 3A). The three *nurf-1* suppressor mutations recovered the fertility of *let-418(L781F)* mutant with various levels (Figure 3B; Supplemental Figure S4A). By measuring brood size of three strains carrying only *nurf-1* mutations at 26°C (Supplemental Figure S4C), we found that all three *nurf-1* mutants displayed no difference in brood size compared with wild-type worms, and we did not detect any apparent abnormalities at 20 or 26°C.

To further validate the suppressor role of the dysfunctional NURF complex, we performed RNA interference (RNAi) to knock down the *nurf-1* or *isw-1* genes, which encodes the *C. elegans* ISWI ATPase in the NURF complex. Both RNAi treatments were able to rescue the sterility of *let-418(L781F)* mutant at 26°C, with *nurf-1* RNAi demonstrating a better rescue efficiency (Figure 3C; Supplemental Figure S4B). These results provide additional evidence that mutations in the NURF complex can rescue the *let-418(L781F)* phenotypes.

### Mutation in the *nurf-1* gene restored chromatin accessibility of *let-418(L781F)* mutant

**FIGURE 4:**
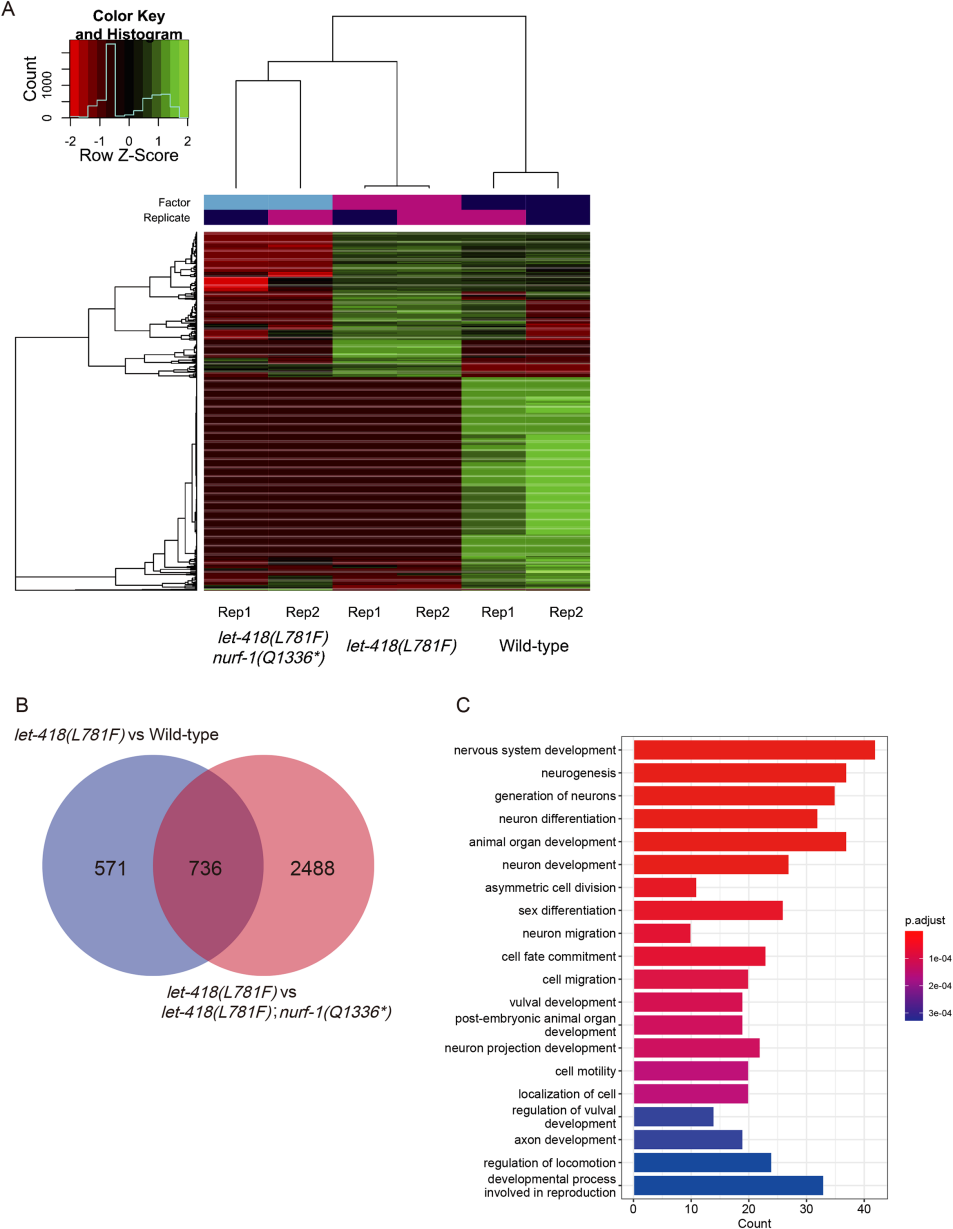
Mutations in the *nurf-1* gene restore chromatin accessibility of *let-418(L781F)* mutant. (A) Heatmap of genes with significant ATAC signal changes in indicated strains at 26°C. (B) Overlap of genes with ectopically upregulated ATAC signals in *let-418(L781F)* worms compared with wild-type or *let-418(L781F)*; *nurf-1(*Q1336*) double mutant. (C) GO enrichment analysis of 736 overlapping genes in B. Metascape was used to analyze the enrichment of genes and the Fisher exact test was used for statistical analysis.

To explore how *nurf-1* mutations rescue *let-418(L781F)* mutant, we compared ATAC-seq signals of WT, *let-418(L781F)* and *let-418(L781F)*; *nurf-1(*Q1336*) double mutant at 26°C. We found that genes exhibiting prominent changes in chromatin accessibility could be classified into two groups. One group displayed upregulated accessibility specifically in *let-418(L781F)* mutant, suggesting the chromatin accessibility of this group of genes have been restored by *nurf-1(*Q1336*), and the other group showed downregulated accessibility in both *let-418(L781F)* mutant and the *let-418(L781F)*; *nurf-1(*Q1336*) double mutant (Figure 4A). We also noticed a cluster of genes at the top of the heatmap showing positive values for both WT and mutant but negative values for the double mutants (Figure 4A). When comparing the WT and mutant worms in the heatmap, these genes exhibited increased chromatin accessibility in the mutant worms. While in the double mutant worms, the chromatin accessibility of these genes decreased to a level lower than that observed in the WT worms. Consequently, we continue to categorize these genes as displaying upregulated accessibility specifically in the *let-418(L781F)* mutant, as they exhibited similar trends across different strains. It is conceivable that the upregulation of these genes may contribute to abnormal phenotypes in *let-418(L781F)* worms, while downregulation of these genes exerts only minor effects on development.

Further analysis of the genes with upregulated chromatin accessibility in *let-418(L781F)* mutant revealed that 736 genes were upregulated in *let-418(L781F)* mutant when comparing with either WT or *let-418(L781F)*; *nurf-1(*Q1336*) double mutant (Figure 4B). Gene ontology (GO) term analysis of the 736 overlapping genes revealed that they are enriched in various developmental pathways (Figure 4C), which suggests that *nurf-1* mutations suppress the *let-418(L781F)* mutant by restoring normal accessibility to the promoters of multiple developmental genes. Supplemental Table S2 lists the genes with open chromatin that may lead to observed abnormal phenotypes of *let-418(L781F)* worms.

## DISCUSSION

This study utilized *C. elegans* to model one of the clinical variants of the NuRD subunit CHD-3, specifically the L915F mutation in neurodevelopmental disorders. We show that the L915F defects can be restored by intragenic mutations that likely restore proper NuRD activity. We also show that inhibition of NURF, which is considered as the NuRD’s antagonistic factor, rescued the lethality of NuRD carrying L915F. We provide evidence that the coordinated action of NuRD and NURF is essential for the proper transcriptome and animal viability. These results suggest that NURF might be a potential target for intervening in NuRD-associated diseases.

Unlike many other disease-related mutations in CHD-3 that resulted in impaired activity, in vitro biochemical assays show that the L915F mutant protein exhibited hyperactivity (Snijders Blok *et al.*, 2018). However, patients harboring the L915F variant displayed phenotypes similar to ones carrying loss-of-function mutations (Snijders Blok *et al.*, 2018). Consistent with this, *let-418(L781F)* mutant in *C. elegans* exhibited similar phenotypes to a null allele, including sterility and vulva abnormalities (von Zelewsky *et al.*, 2000), indicating the phenomenon that hyperactive CHD-3 phenocopies null alleles is conserved across species. In support of the phenotypes, our ATAC-seq data revealed a more open chromatin structure in *let-418(L781F)* mutant, suggesting an impaired activity of LET-418 in vivo, as the NuRD complex has been documented for its role in upregulating nucleosome density around promoter regions (Clapier *et al.*, 2017). This implies an unknown mechanism that restricts the activity of hyperactive chromatin-remodeling factors in vivo, thereby generating loss-of-function defects in mutant animals. Our previous study demonstrated that hyperactive kinases can be restricted by RNA editing on their own mRNA molecules in vivo (Li *et al.*, 2021). We examined RNA editing at the *let-418* locus in *let-418(L781F)* mutant but did not observe any apparent RNA editing events (Supplemental Figure S5), suggesting an RNA editing independent regulation might be involved. Further studies are required to elucidate the mechanism underlying the restriction of hyperactive chromatin-remodeling factors in live animals.

The precise mechanism by which a single amino-acid substitution, such as L915F in CHD-3, can dramatically alter chromatin remodeling activity is intriguing. Utilizing Alphafold2-based computational methods, we predicted the structures of human CHD3, relaxed these structural models using Rosetta (Chaudhury *et al.*, 2010), and aligned them with a previously published CHD4 structure bound to a nucleosome (Farnung *et al.*, 2020; Supplemental Figure S6A). In comparison to the nucleosome-bound CHD4, the wild-type CHD3 revealed a relatively exposed DNA binding site, suggestive of an induced conformational adaptation when CHD3 engages with the nucleosome. In contrast, the L915F mutant of CHD3 exhibited a narrower DNA binding site compared with the wild-type CHD3 (Supplemental Figure S6B), which hints at a potentially heightened affinity between CHD3 and the nucleosome, subsequently resulting in hyperactivity. Similarly, the L781F mutation in the worm LET-418 also conferred a narrowed DNA binding site in comparison to the wild-type protein, suggesting a similar hyperactivity of the mutant protein in *C. elegans* (Supplemental Figure S6C).

Through our suppressor screens, we identified intragenic suppressor mutations in *let-418* that were able to restore normal activity. Most of these suppressor mutations were found adjacent to the L781 site, suggesting that they might restore the normal function of LET-418 by direct interaction with L781F. However, two mutations, A984T and D986N, were located relatively far from the L781 site. These mutations may restore LET-418 activity through indirect interactions. It is worth noting that two intragenic suppressor mutations, G827R and D986D, correspond to residues that are mutated in Snijders Blok-Campeau syndrome (G961 and D1120 in human CHD3). In an effort to elucidate the structural consequences of these mutations on LET-418, we conducted structural predictions for LET-418(L781F, G827R). The G827R mutation was observed to broaden the DNA binding site of the LET-418(L781F) protein, potentially leading to the mitigation of the hyperactivity exhibited by LET-418(L781F; Supplemental Figure S6C).

Our investigation of intergenic suppressors has unveiled a delicate equilibrium between distinct chromatin-remodeling factors: defects incurred by a mutation in one remodeling factor, NuRD, can be rescued through a mutation in a counteractive remodeling factor, NURF. In organisms with normal chromatin dynamics, we propose that NuRD and NURF complexes operate akin to a metaphorical seesaw. These two remodeling factors intricately bind to the same promoter, and their opposing activities necessitate careful balancing to establish appropriate chromatin architecture conducive to gene expression. This interplay of activities may likely represent a prevalent phenomenon among various chromatin-remodeling factors. We speculate that mutations in other remodeling factors might also exhibit the capacity to rescue the developmental impairments observed in *let-418(L781F)* mutant.

We did not detect strong correlation between upregulated chromatin accessibility and RNA expression. We speculate that LET-418(L781F) caused abnormal open chromatin of a subset of developmental genes, while these genes further regulated transcriptome in mutant worms and finally contributed to abnormal phenotypes.

Directly targeting CHD-3 itself may not present a viable therapeutic option for the treatment of neurodevelopmental syndromes, given the heterogeneous impact of different clinical variants on CHD-3. Drugs designed to specifically address a particular clinical variant may prove ineffective against other variants. However, our findings regarding the role of NURF offer an alternative avenue for the potential treatment of CHD-3 mutation-related syndromes. Patients could potentially benefit from therapeutic interventions aimed at suppressing NURF activity during critical stages of development. Such targeted treatment strategies might have the potential to be effective across various clinical variants of CHD-3.

## MATERIALS AND METHODS

### Worm strains and culture

*C. elegans* were cultured according to the standard methods (Brenner, 1974). Worms were cultivated on nematode growth medium (NGM) plates seeded with the *Escherichia coli* OP50 unless stated otherwise. The standard cultivation temperature is 20°C unless stated otherwise. The wild-type strain was Bristol N2. Some strains were generated by SunyBiotech using CRISPR-Cas9, while some strains were cloned from forward genetic screen of *let-418(L781F)* strain. Supplemental Table S1 summarizes the strains used in this study.

### EMS mutagenesis and suppressor screen

We used forward genetic screens to isolate suppressors of *let-418(L781F)* strain. The *let-418(L781F)* mutant worms (P0) were synchronized to the late L4 larval stage, washed off from plates and collected in 4 ml M9 buffer, and incubated with 50 mM ethyl methanesulfonate (EMS) for 4 h at room temperature with constant rotation. Worms were then washed with M9 three times and cultured on standard NGM plates. After about 24 h, adult animals were bleached to collect eggs. Eggs (F1) were cultured under standard conditions until F1 worms began laying eggs. All NGM plates were then transferred to and cultivated under 26°C. Fertile worms were individually cultured, and fertility of their progenies was further examined under 26°C. We identified mutations using whole-genome sequencing.

### RNA sequencing

Embryos were mainly used for RNA-seq. To collect embryos, synchronized adults are collected and incubated in bleach buffer until most adults are lysed. For each sample, embryos were lysed in 400 µL TRIzol reagent (Invitrogen). Total RNA was extracted according to the manufacturer’s protocol. To prevent RNA degradation, SUPERase•In^TM^ RNase Inhibitor (Invitrogen) was used in each step. RNA concentration was quantified with the Qubit RNA High Sensitivity Assay Kit (Invitrogen), and RNA quality was measured by the Agilent 2100 bioanalyzer system. RNA samples with an RNA integrity number (RIN) over 6.0 were used to construct cDNA library. 50 ng–500 ng of total RNA was used for library preparation using the KAPA RNA HyperPrep Kit (KAPA Biosystems). Qubit was used to measure library concentration and Agilent 2100 bioanalyzer system was used for quality control. Samples passed quality control were sequenced on an Illumina HiSeq platform.

### Differential gene expression analysis

The raw reads from RNA-seq were assessed for quality, adaptor content, and duplication rates with FastQC. The raw sequencing reads were trimmed using Trim_galore (version 0.4.4) to remove the low-quality bases and adaptor sequences. Paired-end reads with at least 20 nucleotides long were aligned to *C. elegans* reference genome (ce11) using STAR (2.5.4 b). The numbers of reads that aligned to genes were quantified by HTSeq (version 0.9.1). Only uniquely mapped reads were counted into the relative expression level of the gene. Reads aligned to multiple genes multiple regions were excluded. Gene names were annotated in the DAVID website (https://david.ncifcrf.gov/). Differentially expressed genes were identified using DESeq2 package in R programming language. Genes with false discovery rate (FDR) [lt] 0.05, log2-transformed fold change greater than 1, un-normalized counts from HTSeq greater than 3 were selected as upregulated genes; genes with FDR [lt] 0.05, log2-transformed fold change [lt] -1, unnormalized counts greater than three were selected as downregulated genes; other genes were identified as not differentially expressed. ggplot2 package in R programming language was used to plot figures.

### Extracting genomic DNA

Worms are cultured on NGM plates, washed off by M9 buffer, and washed by TEN buffer (20 mM pH8.0 Tris-HCl, 50 mM ethylenediaminetetraacetic acid (EDTA), 100 mM NaCl). Pelleted worms were resuspended in 500 µl TEN buffer with 0.5% sodium dodecyl sulfate and 200 µl/ml proteinase K. Each tube was incubated at 65°C for 2 hours, mixed occasionally and added proteinase K every hour. 500 μL PCI buffer was used to extract DNA, and DNA was precipitated by 1.25 ml cold pure ethyl alcohol and washed by 75% ethyl alcohol for 3 times. DNA was dissolved in 500 μl TEN buffer and added 3 μl of 10 mg/mL RNase A, incubated at 37°C for 0.5 hour to remove RNA. PCI extraction is performed as before, and DNA pellet is dissolved in 50 μl ddH2O.

### ATAC-seq

Embryos were also used for ATAC-seq, and worm embryos are collected as same as RNA-seq. Specifically, to collect embryos at 26°C, L4 worms were transferred to 26°C for 1 d and bleached to release embryos. The live embryos are then incubated in chitinase solution and slowly rotated at room temperature for 0.5–1 h to digest egg shells. Digested embryos are pelleted and resuspended in egg buffer. embryos were dissociated into single cells by pipetting up and down for 70–100 times. Embryo cells were pelleted by low-speed centrifugate and resuspended in ATAC lysis buffer (Wu *et al.*, 2018), incubated at 4°C for 10 min to break cell membrane and release nuclei. Nuclei were used for library preparation directly by Vazyme TruePrep DNA Library Kit (Vazyme). Genomic DNA was used as input for negative control. The libraries are sent to Novogene for high-throughput sequencing. Qubit was used to measure library concentration and Agilent 2100 bioanalyzer system was used for quality control. Samples passed quality control were sequenced on an Illumina HiSeq platform.

### Peak calling

The raw reads from ATAC-seq were assessed for quality, adaptor content, and duplication rates with FastQC. The raw sequencing reads were trimmed using Trim_galore (version 0.4.4) to remove the low-quality bases and adaptor sequences. Paired-end reads with at least 20 nucleotides long were aligned to *C. elegans* reference genome (ce11) using bwa(version 0.7.17). macs2(version 2.1.1) was used to call peaks of short DNA fragments. Peaks were annotated to genes by ChIPseeker package in R programming language. Differential peaks were identified by DiffBind package in R programming language. ggplot2 package in R programming language was used to plot figures.

### Sequence alignment

Protein sequences were obtained from Wormbase (www.wormbase.org/) or National Center for Biotechnology Information database (www.ncbi.nlm.nih.gov/). Sequence alignment was performed by Clustal X2.1 (www.clustal.org/). Sequences used for alignment include CE17716 (*Ce* LET-418), CE03657 (*Ce* CHD-3), NP_001005273.1 (*Hs* CHD3), NP_649111.1 (*Ds* CHD3), and CAA99517.1 (*Sc* SNF2). Full-length sequences were used to perform alignment.

### Brood size counting

To count brood size of each strain, individual late L4 stage hermaphrodites were picked to separate plates and transferred daily to new plates until no more eggs were laid. Live progeny were counted at the late larva to adult stage.

### RNA interference

RNAi of worms was performed by feeding assay. The RNAi clones were from the Ahringer *C. elegans* library and each clone was confirmed by sanger sequencing. Bacteria from RNAi clones were cultured in liquid LB medium plus carbenicillin at 50 µg ml^-1^ and tetracycline at 12.5 µg ml^-1^ overnight, and then seeded on NGM plates containing 50 ng ml^-1^ carbenicillin, 12.5 µg ml^-1^ tetracycline, and 1 mM isopropyl β-d-1-thiogalactopyranoside (IPTG). For negative control, bacteria containing empty vector L4440 were cultured and seeded as RNAi clones.

### Imaging

Adult *C. elegans* hermaphrodites were anesthetized with 0.1 mmol/L levamisole in M9 buffer, mounted on 3% agarose pads, and maintained at room temperature for imaging. Imaging was performed using a Zeiss Axio Observer Z1 microscope equipped with 405, 488, and 561 laser lines, a Yokogawa spinning disk head, an Andor iXon+ EM-CCD camera, and a Zeiss 100 × /1.46 objective. Images were acquired by µManager (www.micromanager.org). All the images were taken using identical settings. Image analysis and measurement were performed with ImageJ software (http://rsbweb.nih.gov/ij/). Image stacks were z-projected using maximum projection.

### Data reporting and statistics

The sample sizes of our experiments were determined from related published analyses. GraphPad Prism 7 (GraphPad Software) was used for statistical analyses. Independent Student’s *t* tests were performed to compare the mean values between two groups. Statistic significances were designated as: *p < 0.05; **p < 0.01; and ***p < 0.001. The information about statistical tests, *P* values, and *n* numbers was provided in the respective figures and figure legends.

## Supplementary Material



## Abbreviations used:

ATAC-seq: Assay for Transposase-Accessible Chromatin with high-throughput sequencing
EDTA: ethylenediaminetetraacetic acid
GO: gene ontology
IPTG: isopropyl β- d-1-thiogalactopyranoside
NuRD: nucleosome remodeling and deacetylase
NURF: nucleosome remodeling factor
PCA: Principal Component Analysis
RNAi: RNA interference
TSS: transcription start site
WT: wild-type
