# Supplementary material for: Inhibition of the chromatin remodeling factor NURF rescued sterility by a clinic variant of NuRD
Source: Mol Biol Cell. 2023 Dec 18;35(1):ar13. doi: 10.1091/mbc.E23-05-0197 (PMC10881175; doi:10.1091/mbc.E23-05-0197)
Supplement: Supplementary file 1 [file mbc-35-ar13-s001.pdf]

Supplemental Materials

Molecular Biology of the Cell

Shen *et al*.

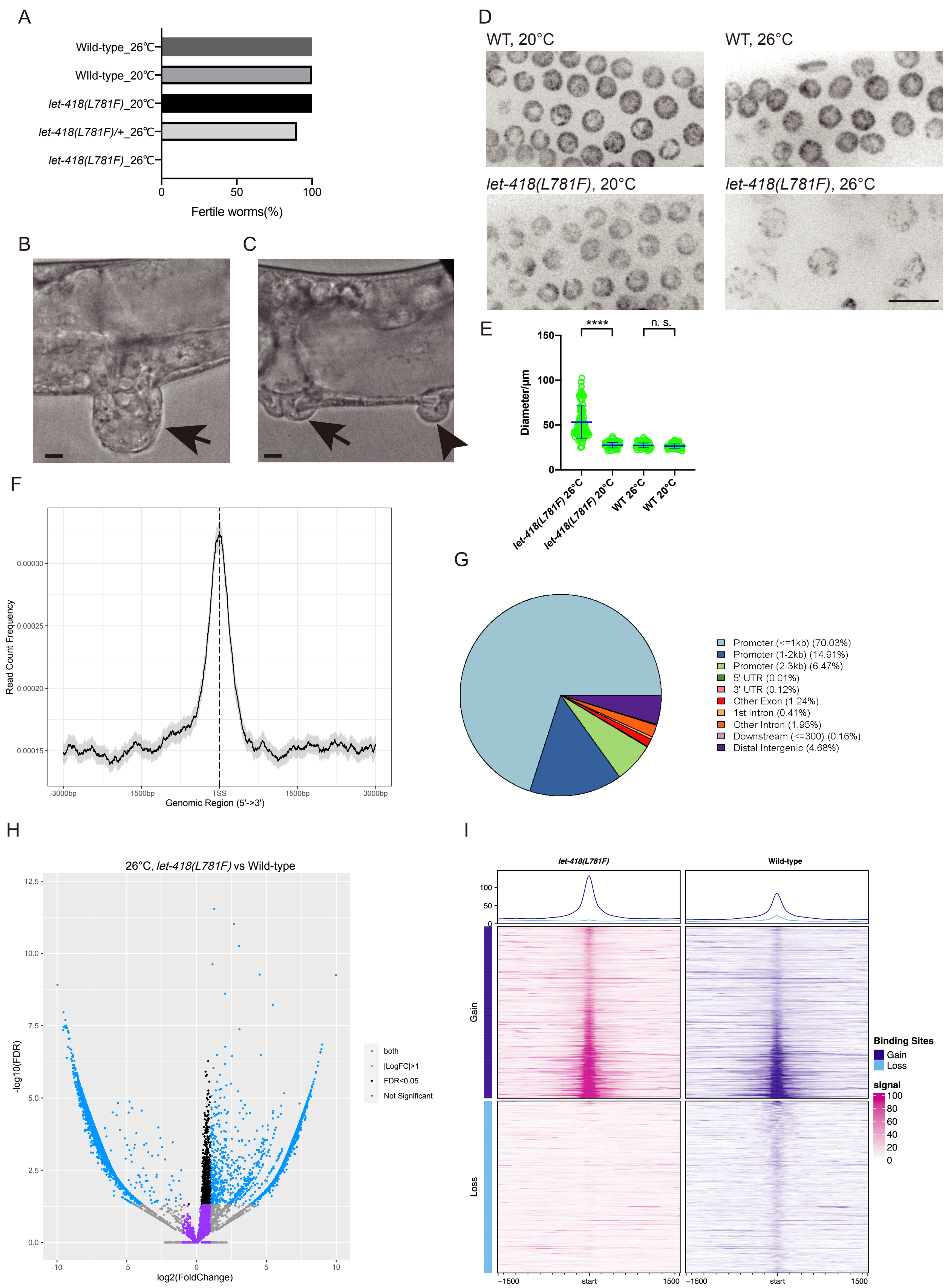
A
Wild-type_26℃
WIld-type_20℃
let-418(L781F)_20℃
let-418(L781F)/+_26℃
let-418(L781F)_26℃
Fertile worms(%)
B
C
D
WT, 20°C
WT, 26°C
let-418(L781F), 20°C
let-418(L781F), 26°C
E
Diameter/μm
****
n. s.
let-418(L781F) 26°C
let-418(L781F) 20°C
WT 26°C
WT 20°C
F
Read Count Frequency
Genomic Region (5'->3')
TSS
G
Promoter (<=1kb) (70.03%)
Promoter (1-2kb) (14.91%)
Promoter (2-3kb) (6.47%)
5' UTR (0.01%)
3' UTR (0.12%)
Other Exon (1.24%)
1st Intron (0.41%)
Other Intron (1.95%)
Downstream (<=300) (0.16%)
Distal Intergenic (4.68%)
H
26°C, let-418(L781F) vs Wild-type
-log10(FDR)
log2(FoldChange)
both
|LogFC|>1
FDR<0.05
Not Significant
I
let-418(L781F)
Wild-type
Gain
Loss
Binding Sites
signal
start

Figure S1. Sterility, vulva defects and abnormal chromatin accessibility in disease model *let-418(L781F)* worm at 26 °C.

(A) The percentage of fertile animals of wild-type and *let-418(L781F)* at 20°C or 26 °C. N=10. **(B)** Protruding vulva of *let-418(L781F)* mutant at 26 °C. Arrow indicates the abnormal protruding vulva. **(C)** Multivulva phenotype of *let-418(L781F)* mutant under 26 °C. Arrow indicates the abnormal protruding vulva; arrow head indicates the pseudo vulva. Scale bar = 10 μm. **(D)** Nuclei of *C. elegans* germ cells in wild-type and *let-418(L781F)* mutant at 20 °C and 26 °C. Wild-type LET-418 or LET-418(L781F) is labeled with GFP. **(E)** Diameter of germ cell nuclei in (C). N=100, values are presented as mean ± SD (error bars). Statistical significance is based on Student's t-test, n.s., not significant, *$p< 0.05$, **$p< 0.01$, ***$p< 0.001$, ****$p< 0.0001$. Scale bar = 10 μm. **(F)** The average of Read Count Frequency from ATAC-seq at promoter regions of all genes. **(G)** Peak distributions of ATAC signals in different genome regions. **(H)** Volcano plot comparing ATAC signals of each gene between wild-type and *let-418(L781F)* at 26 °C. **(I)** The average and heatmap of ATAC-seq signals at promoter regions with significant changes (|Fold Change| > 2) between wild-type and *let-418(L781F)* at 26 °C.

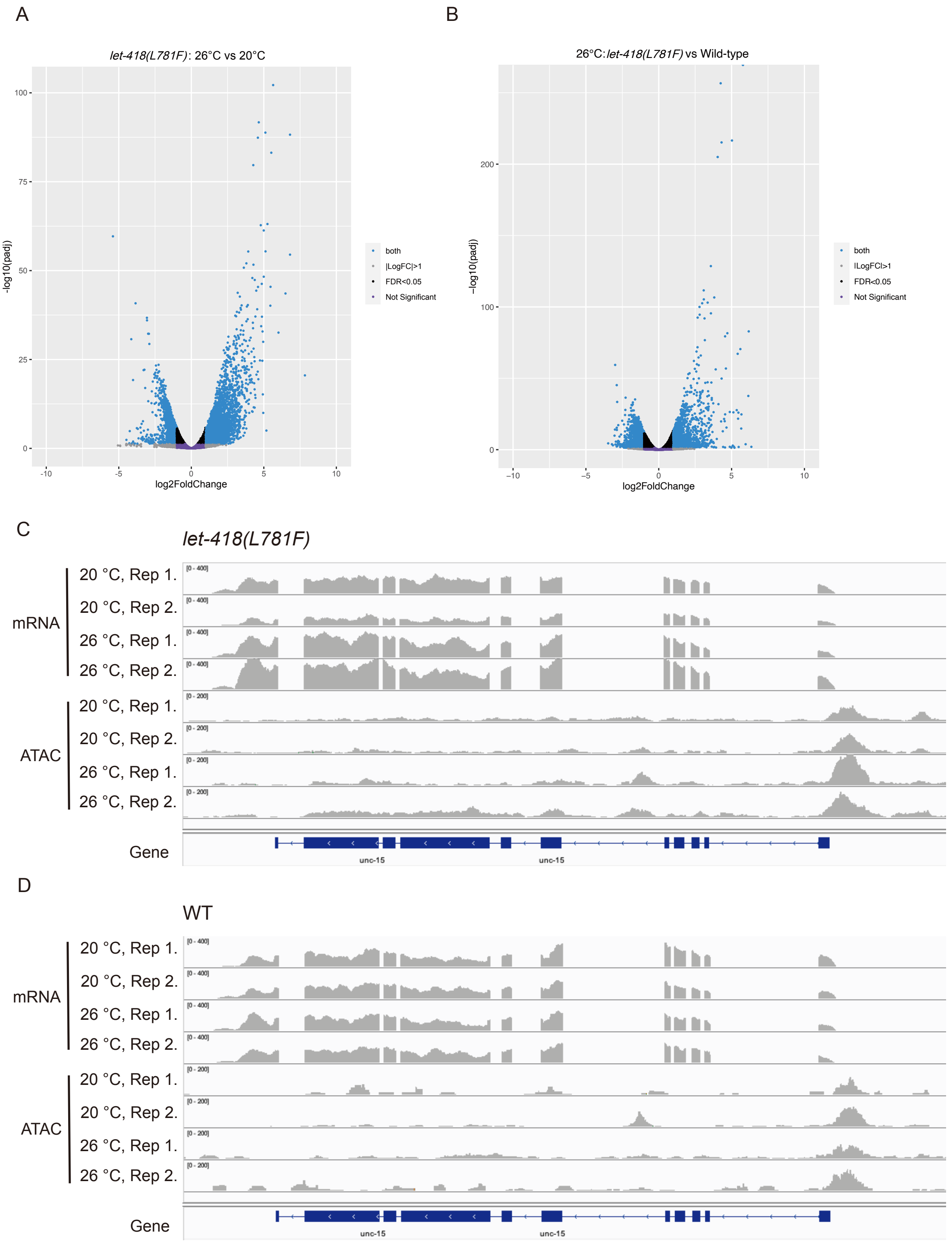

A
let-418(L781F): 26°C vs 20°C
-log10(padj)
100
75
50
25
0
-10
-5
0
5
10
log2FoldChange
both
|LogFC|>1
FDR<0.05
Not Significant
B
26°C:let-418(L781F) vs Wild-type
−log10(padj)
200
100
0
−10
−5
0
5
10
log2FoldChange
both
|LogFC|>1
FDR<0.05
Not Significant
C
let-418(L781F)
mRNA
ATAC
20 °C, Rep 1.
20 °C, Rep 2.
26 °C, Rep 1.
26 °C, Rep 2.
20 °C, Rep 1.
20 °C, Rep 2.
26 °C, Rep 1.
26 °C, Rep 2.
[0 - 400]
[0 - 200]
Gene
unc-15
unc-15
D
WT
mRNA
ATAC
20 °C, Rep 1.
20 °C, Rep 2.
26 °C, Rep 1.
26 °C, Rep 2.
20 °C, Rep 1.
20 °C, Rep 2.
26 °C, Rep 1.
26 °C, Rep 2.
[0 - 400]
[0 - 200]
Gene
unc-15
unc-15

Figure S2. Abnormal transcriptome in *let-418(L781F)* mutant.

(A) Volcano plot comparing mRNA abundance of each gene in *let-418(L781F)* at 20 °C and 26 °C. **(B)** Volcano plot comparing mRNA abundance of each gene between wild-type and *let-418(L781F)* mutant at 26 °C. **(C)** Distributions of normalized RNA-seq and ATAC-seq reads of a representative gene (*unc-15*) in *let-418(L781F)* mutant. **(D)** Distributions of normalized RNA-seq and ATAC-seq reads of a representative gene (*unc-15*) in wild-type worms.

A

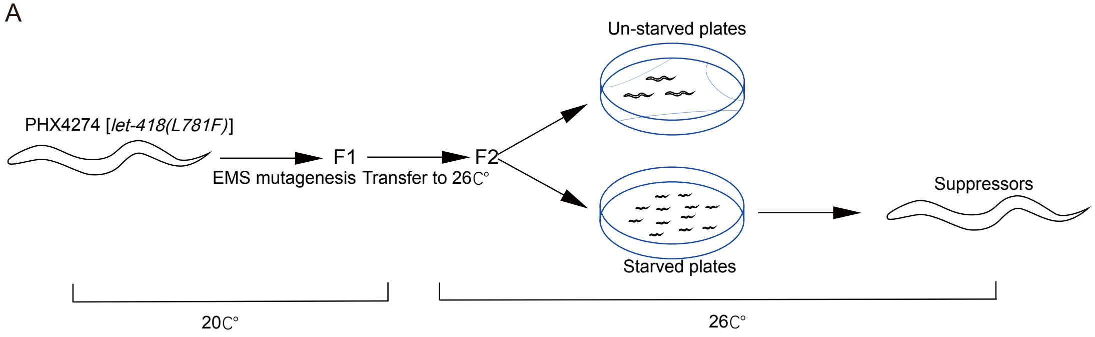

B

Intragenic suppressor strain	Mutation
cas1710	L792F
cas1709, cas1721	S814F
cas1717, cas1720	L822F
cas1718, cas1730	G827R
cas1702, cas1719	M830I
cas1713, cas1716	R832H
cas1723	R832C
cas1722	L834F
cas1714	A984T
cas1725	D986N

C

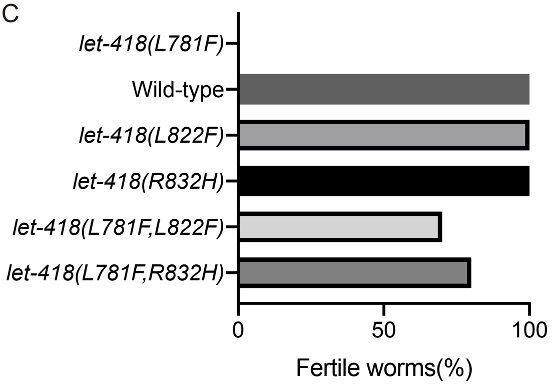

D

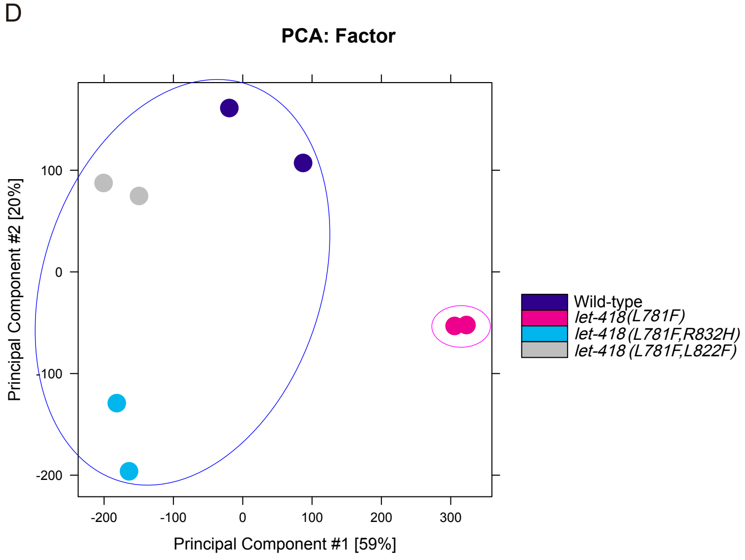

Figure S3. Intragenic mutations in *let-418* restore fertility and chromatin accessibility of *let-418(L781F)*.

(A) Schematic of suppressor screen of *let-418(L781F)* mutant. **(B)** Independent intragenic suppressor strains and the residue mutations in LET-418. **(C)** Brood size of the indicated strains under 26 °C. N=10. **(D)** PCA analysis of ATAC-seq data in wild-type, *let-418(L781F)*, *let-418(L781F,R832H)* and *let-418(L781F,L822F)*. *let-418(L781F,R832H)* and *let-418(L781F,L822F)* clustered with wild-type worms, and *let-418(L781F)* clustered alone.

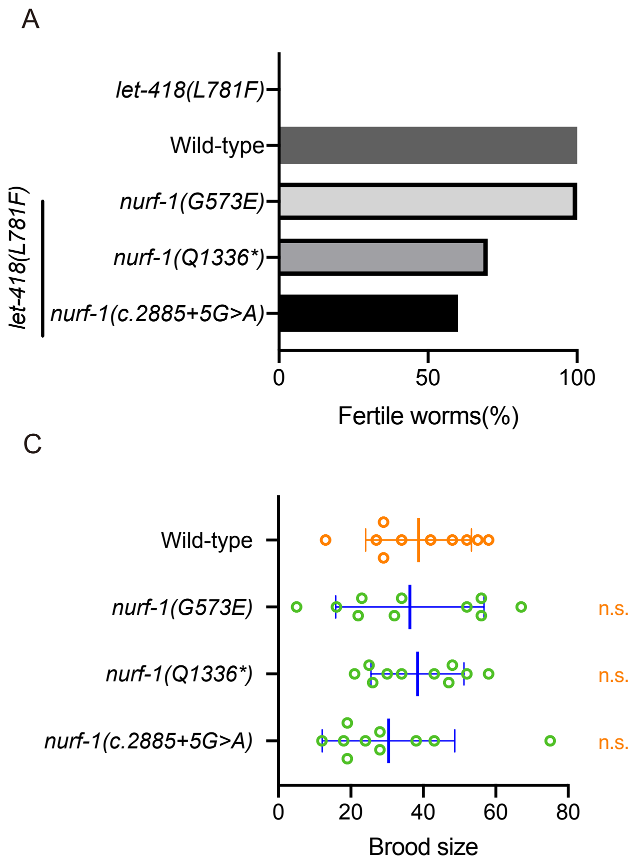

A
let-418(L781F)
Wild-type
nurf-1(G573E)
nurf-1(Q1336*)
nurf-1(c.2885+5G>A)
let-418(L781F)
0
50
100
Fertile worms(%)
B
control RNAi
control RNAi
nurf-1 RNAi
isw-1 RNAi
let-418(L781F)
0
50
100
Fertile worms(%)
C
Wild-type
nurf-1(G573E)
nurf-1(Q1336*)
nurf-1(c.2885+5G>A)
n.s.
n.s.
n.s.
0
20
40
60
80
Brood size

Figure S4. Intergenic mutations in the *nurf-1* gene restore *let-418(L781F)* fertility.

(A) Brood size of the indicated strains at 26 °C. N=10. **(B)** Brood size of the indicated RNAi strains at 26 °C. N=10. **(C)** Brood size of the indicated strains at 26 °C. N=10, values are presented as mean ± SD (error bars). Statistical significance compared with the control with a matching color code is based on Student's t-test, n.s., not significant.

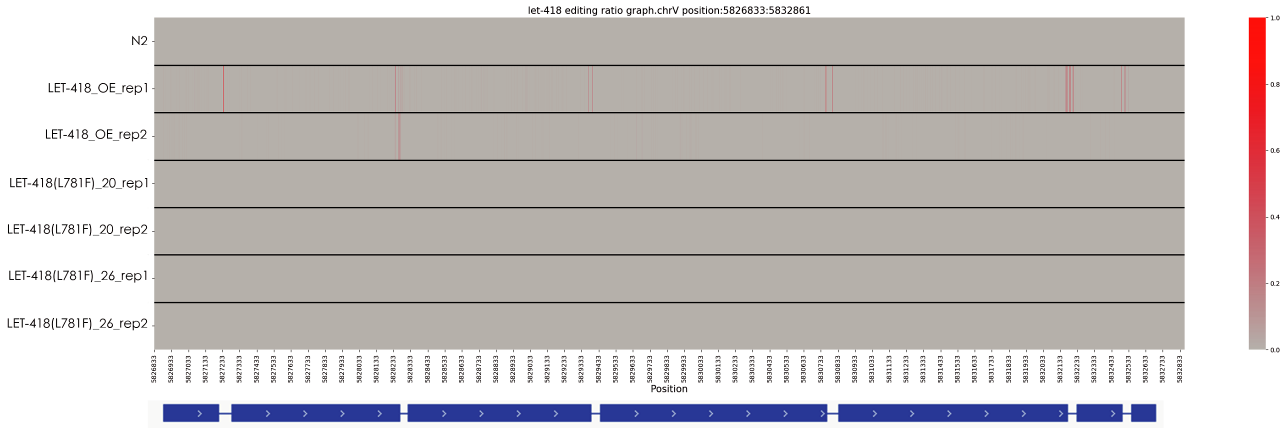

let-418 editing ratio graph.chrV position:5826833:5832861
N2
LET-418_OE_rep1
LET-418_OE_rep2
LET-418(L781F)_20_rep1
LET-418(L781F)_20_rep2
LET-418(L781F)_26_rep1
LET-418(L781F)_26_rep2
Position

Figure S5. ***let-418(L781F)*** **displays no apparent RNA-editing on** ***let-418*** **locus**

Representative RNA editing analysis at the *let-418* locus in the indicated strains under certain temperature. Each row corresponds to a sample. Red lines represent RNA editing sites in the genomic locations of *let-418*,and the bar for editing level is shown in the right.

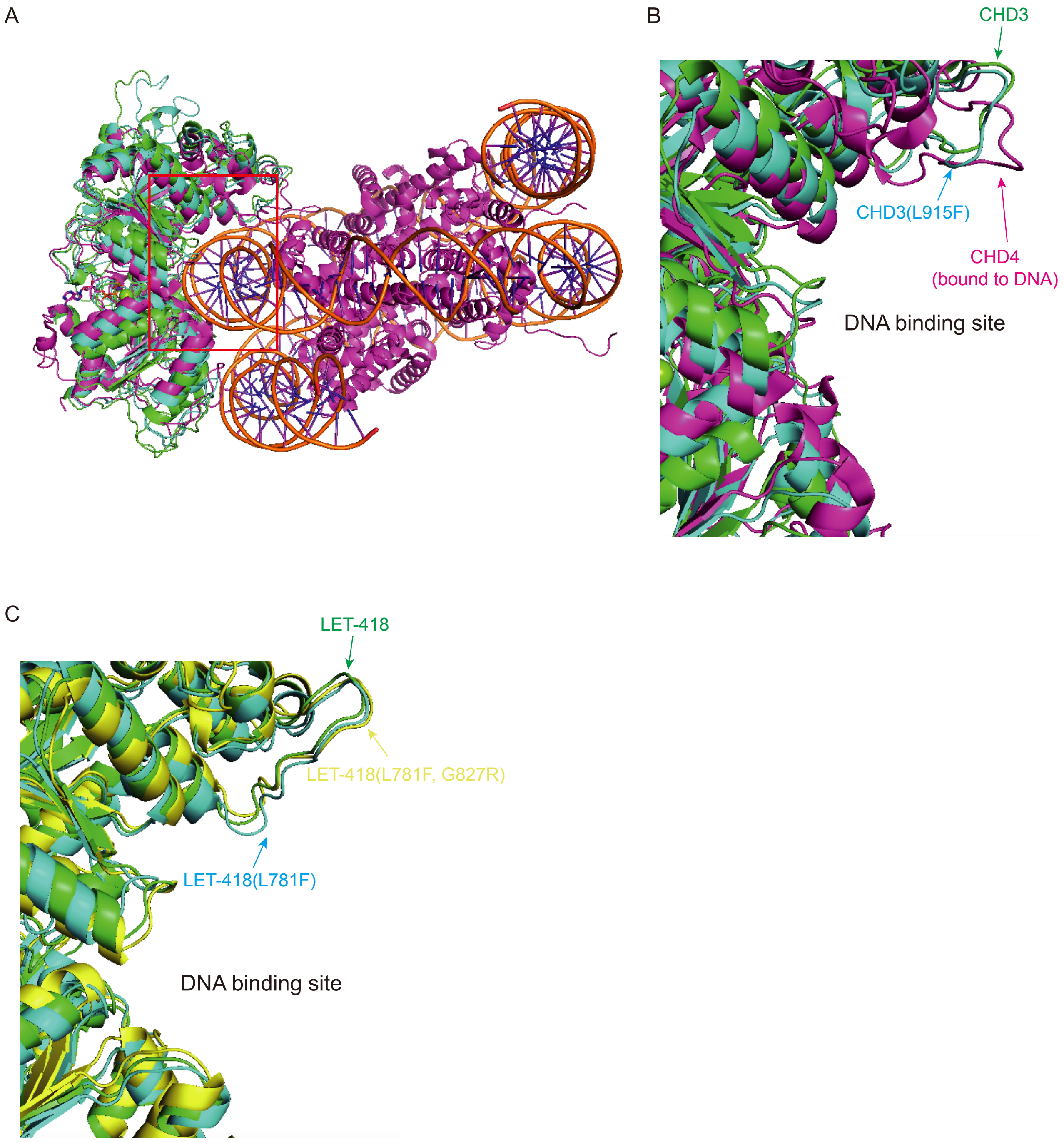

A
B
CHD3
CHD3(L915F)
CHD4
(bound to DNA)
DNA binding site
C
LET-418
LET-418(L781F, G827R)
LET-418(L781F)
DNA binding site

Figure S6. Possible impacts of mutations on CHD3/LET-418 structures

(A) Human CHD3 structures predicted by Alphafold2 and relaxed by Rosetta. Structures were aligned to CHD4 structure in nucleosome-binding state. **(B)** Zoom-in view of the DNA binding site of wild-type human CHD3, CHD3(L915F) and CHD4 bound to nucleosome. (C) Zoom-in view of the DNA binding site of wild-type LET-418, LET-418(L781F), LET-418(L781F, G827R) in *C. elegans*.

Table S1 Worm strains of this study

Strain name	Genotype
N2	Wild-type
PHX4274	*let-418(L781F)*
PHX5589	*let-418(R832H)*
PHX5624	*let-418(L822F)*
cas1713	*let-418(L781F, R832H)*
cas1715	*let-418(L781F); nurf-15(Q1336*)*
cas1717	*let-418(L781F, L822F)*

Table S2 Possible genes contributed to phenotypes of *let-418(L781F)* strain

GO term ID	Description	Genes with up-regulated accessibility at 26°C
GO:0007399	nervous system development	*ect-2/fmi-1/pat-3/die-1/unc-2/hlh-16/cog-1/cwn-1/sox-2/mig-2/cnt-2/hlh-2/unc-37/unc-73/hmr-1/goa-1/par-1/ida-1/kal-1/lin-9/tns-1/mig-10/cfz-2/ceh-5/ceh-40/egl-46/sax-2/ref-2/ngn-1/vab-3/T27C4.1/inx-19/ztf-11/dex-1/unc-76/cam-1/epi-1/dma-1*
GO:0007548	sex differentiation	*cnnm-1/vav-1/ani-1/cdh-3/F42F12.3/rsp-1/cki-2/cog-1/sex-1/riok-1/mig-2/ksr-2/hlh-2/ver-3/lin-15B/unc-73/xnp-1/kal-1/lin-9/sys-1/lag-2/cyd-1/cogc-3/vab-3/dmd-9/sdc-2*
GO:0040025	vulval development	*cnnm-1/vav-1/pat-3/cwn-1/riok-1/ksr-2/hlh-2/lin-15B/unc-73/xnp-1/cdc-14/par-1/lin-9/mig-10/lag-2/sem-2/let-418/lin-13*
GO:0003006	developmental process involved in reproduction	*cnnm-1/emb-8/vav-1/ani-1/mes-1/xol-1/cdh-3/F42F12.3/rsp-1/cki-2/cog-1/sex-1/riok-1/mig-2/ksr-2/hlh-2/ver-3/lin-15B/unc-73/xnp-1/atz-1/kal-1/lin-9/sys-1/lag-2/cyd-1/cogc-3/vab-3/her-1/dmd-9/sdc-2/ubxn-3*
